# Nonlinear Rydberg exciton-polaritons in Cu_2_O microcavities

**DOI:** 10.1038/s41377-024-01382-9

**Published:** 2024-02-06

**Authors:** Maxim Makhonin, Anthonin Delphan, Kok Wee Song, Paul Walker, Tommi Isoniemi, Peter Claronino, Konstantinos Orfanakis, Sai Kiran Rajendran, Hamid Ohadi, Julian Heckötter, Marc Assmann, Manfred Bayer, Alexander Tartakovskii, Maurice Skolnick, Oleksandr Kyriienko, Dmitry Krizhanovskii

**Affiliations:** 1 Department of Physics and Astronomy, University of Sheffield, Sheffield, S3 7RH UK; 2 Department of Physics and Astronomy, University of Exeter, Stocker Road, Exeter, EX4 4PY UK; 3 SUPA, School of Physics and Astronomy, University of St Andrews, St Andrews, KY16 9SS UK; 4 Fakultät Physik, TU Dortmund, August-Schmidt-Straße 4, 44227 Dortmund, Germany

**Keywords:** Microresonators, Nonlinear optics, Polaritons

## Abstract

Rydberg excitons (analogues of Rydberg atoms in condensed matter systems) are highly excited bound electron-hole states with large Bohr radii. The interaction between them as well as exciton coupling to light may lead to strong optical nonlinearity, with applications in sensing and quantum information processing. Here, we achieve strong effective photon–photon interactions (Kerr-like optical nonlinearity) via the Rydberg blockade phenomenon and the hybridisation of excitons and photons forming polaritons in a Cu2O-filled microresonator. Under pulsed resonant excitation polariton resonance frequencies are renormalised due to the reduction of the photon-exciton coupling with increasing exciton density. Theoretical analysis shows that the Rydberg blockade plays a major role in the experimentally observed scaling of the polariton nonlinearity coefficient as ∝ *n*^4.4*±*1.8^ for principal quantum numbers up to *n* = 7. Such high principal quantum numbers studied in a polariton system for the first time are essential for realisation of high Rydberg optical nonlinearities, which paves the way towards quantum optical applications and fundamental studies of strongly correlated photonic (polaritonic) states in a solid state system.

## Abstract (web summary)

Strong effective photon–photon interactions (Kerr-like optical nonlinearity) via the Rydberg blockade phenomenon in Cu_**2**_O-microcavity achieved under pulsed resonant excitation enabling fundamental studies of strongly correlated polaritonic states and quantum optical applications.

## Graphical abstract

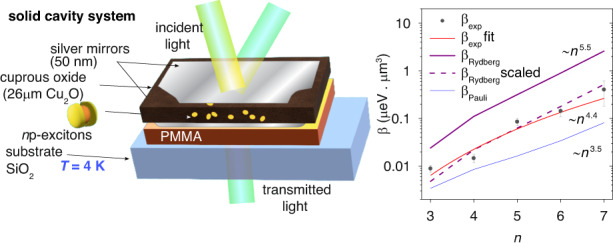

## Introduction

Prior to the study of Rydberg excitons in solids, significant efforts have been devoted to the research of Rydberg atoms—giant atomic states with valence electrons occupying orbits of high energy excited states (with sizes up to tens of micrometres). Rydberg states have been at the focus of fundamental and applied science in areas of metrology^1^, sensing^2,3^, quantum information and simulation^4–6^. Their strong long-range dipole–dipole interactions lead to the Rydberg blockade phenomenon^7–9^, where the presence of one excited atom prevents the excitation of another in its vicinity, at the same frequency. This effect forms the basis for quantum information processing (QIP) with Rydberg atoms^10–13^. Furthermore, coupling light to Rydberg atoms^14,15^ enables strong effective photon–photon interactions down to the single particle level paving the way towards the development of various quantum optical devices (single-photon switches, phase shifters, transistors etc.^16^).

Recently, Rydberg excitons (the analogue of Rydberg atoms in condensed matter systems) were observed in a number of materials including transition metal dichalcogenides (TMDCs)^17^, perovskites^18^, and Cu_2_O^19–22^, where the Rydberg exciton radius reaches a few microns in states with principal quantum numbers as high as *n* = 30^23^ and ultra-high nonlinearities have been reported in CW regime^21,24^. Rydberg exciton blockade was demonstrated experimentally^25^. On the other hand, there is a strong interest in the study of hybridised excitons and photons in microcavities and waveguides, which lead to the realisation of giant Kerr-like optical (polaritonic) nonlinearities^26,27^. These nonlinearities can be exploited for the development of highly nonlinear and scalable optical devices on a chip with possible applications in QIP^28^. Polariton nonlinear phenomena, such as superfluidity, solitons, photon blockade^29,30^ and single-photon phase shifts^28^, to name a few, were investigated mostly for 1 s Wannier-Mott excitons. Nonlinear energy shifts have been reported for Rydberg exciton-polaritons in TMDCs^17,31,32^ and perovskites^18^, but these studies are limited to the first two excited exciton states. By contrast, large principal numbers *n* are available in Cu_2_O and very recently strong exciton-photon coupling was observed in a microcavity with embedded Cu_2_O^33^, where only the linear optical response of Rydberg exciton-polaritons was addressed.

Here, we study yellow series Rydberg exciton-polaritons in a planar FabryPerot microcavity with embedded Cu_2_O thin crystal. We report a strong and ultra-fast nonlinear optical response for these states and demonstrate the highly superlinear scaling of the nonlinearity with the principal quantum number up to *n* = 7. This scaling opens up the potential for ultra-high nonlinearity at the highest numbers (*n* = 30) observed so far in bare Cu_2_O crystals. The nonlinearities are found to be comparable or even exceeding (for *n* ≥ 5) the giant optical Kerr-like nonlinearities observed in other polariton platforms, such as GaAs or hybrid perovskites^34^. Nonlinear indices n_2_ are found to be in the range from 10^−17^ m^2^/W to 4 × 10^−15^ m^2^/W, for *n* = 3 to *n* = 7. In a single pulse experiment, the response time of nonlinearity must be given by the polariton lifetime comparable to the duration of the pulse ∼1 ps. Additional pump-probe measurements reveal that the nonlinearities at *n* = 4 are found to respond within a picosecond rise time and fall within the ~40 ps range. This ultra-fast response is followed by additional nonlinear dynamics rising and falling on density-dependent timescales of order 100 ps to 2 ns. This demonstrates that multiple processes contribute to the nonlinear response. Crucially, our pulsed resonant excitation method significantly reduces the interactions with long-lived ground state excitations and electron-hole plasma and enables us to access the pure, ultra-fast, Rydberg nonlinearity. To complete our study we provide a theoretical model that takes into account contributions from Rydberg and Pauli blockade and semi-quantitatively explains the observed experimental behaviour at high excitation densities.

## Single pulse experiment

### Experiment

**Fig. 1:**
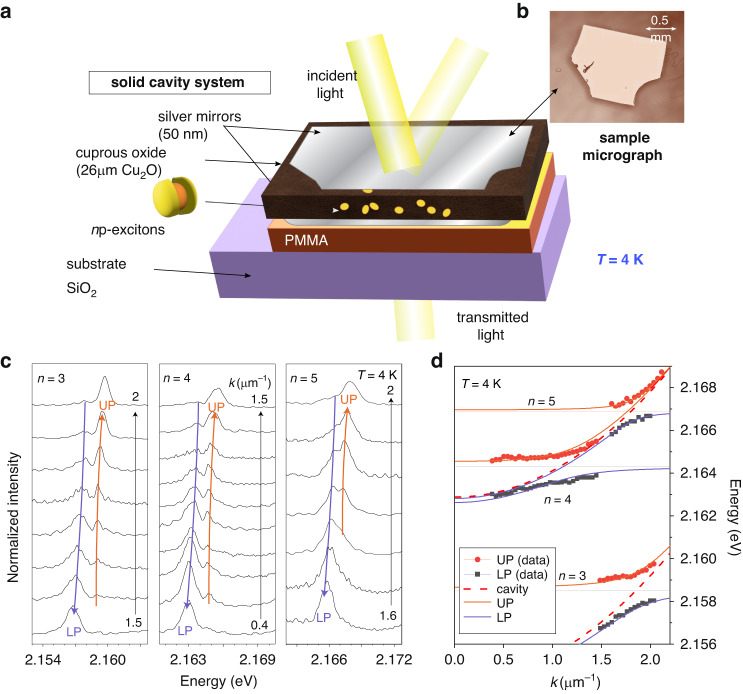
Sketch of the Cu_2_O microcavity sample and its dispersion. **a** A schematic of the solid cavity used for the transmission experiment. **b** Microscopy image of the natural Cu_2_O sample of thickness approximately 26 µm with silver mirrors (recoloured). **c** Waterfall spectra plots taken with super-continuum laser in transmission for different wavevectors near *n* = 3, *n* = 4 and *n* = 5 excitons. Upper and lower polaritons are visible as a doublet at resonant conditions. **d** Polariton peak positions as a function of wavevector extracted from (**c**). Lines show cavity, exciton and polariton dispersions (for higher principal quantum numbers, see SI, Sec. 4)

We study a microcavity system formed by two silver mirrors with an embedded thin flake of Cu_2_O material deposited on top of SiO_2_ substrate with an intermediate PMMA layer (see Fig. 1a, b). Multiple cavity modes with a free spectral range of 9 meV form (see SI, Fig. S1) due to the thickness of Cu_2_O slab of approximately 26 µm. We reveal the strong coupling between the cavity modes and Rydberg excitons in the angular-resolved transmission spectra of a super-continuum laser source (see Fig. 1c), similar to recently published results^33^. The transmission spectra recorded for different in-plane *k*-vectors show anti-crossings between the cavity modes and Rydberg exciton resonances in Fig. 1c with clear doublets corresponding to the upper (UP) and lower (LP) exciton-polariton states. To highlight the strong coupling in Fig. 1d we also plot the dispersion of Rydberg exciton-polaritons for *n* = 3, 4 and 5. The formation of polaritons for *n* = 6 and 7 excitons is not resolved for this position on the sample, since the cavity mode is in resonance at higher wavevectors and the high-frequency spectral noise leads to weak signal-to-noise ratio and in *k*-space prevents observation of the anticrossing. The signal-to-noise ratio is higher for detection in real space (i.e Fourier transform of *k*-space) and the polariton doublets for *n* = 6 and 7 are clearly observed in transmission for a different spot on the sample with a slightly different Cu_2_O thickness, when the energy of the cavity mode at *k* = 0 is tuned into resonance with these excitons by changing position on the sample (SI, Sec. 4).

**Fig. 2:**
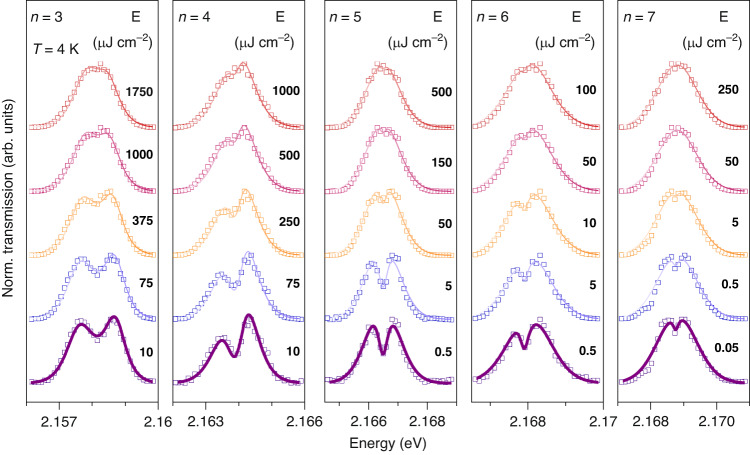
Normalised transmission spectra with increasing pump power. We plot the transmission of the Cu_2_O microcavity from narrowband (1.75 meV) excitation (see Methods) for different principal quantum numbers *n*, taken at different pump fluence *E* = *P*_avg_*/*(*fA*) (*P*_avg_ is pump incident average power, *f* is the repetition rate of the laser, *A* is the effective excitation area). The spectra are normalised to unity and shifted by 1 for clarity. The solid curves represent a fit with a coupled oscillator model

To probe Rydberg exciton-polariton nonlinearities we record the transmission spectra in real space at different energies of the laser pulse (with full width at half maxima FWHM 1.75 meV), directed at normal incidence to the sample and tuned in resonance with the polariton states arising from excitons with different *n*. In Fig. 2 we show the power dependencies of the transmission spectra of these states. At small powers, the doublet of LP and UP states is clearly visible for quantum states from *n* = 3 to *n* = 7. There is a minor asymmetry between the intensities of the lower and upper polariton branches observed for *n* = 4 and 6 states, which is attributed to a small detuning between the laser peak energy and the centre between polariton resonances (this detuning is set manually by the diffraction grating in the pulse shaper and cannot be very precise) so that one resonance is pumped slightly more efficiently than the other.

As power increases the separation between the polariton resonances becomes smaller, which we attribute to the decrease in coupling strength, and eventually a collapse of strong coupling. The resulting transmission spectra profiles become a singlet (here, limited by the pulse spectral width). We note the sharp contrast in power threshold needed to reach a singlet between the *n* = 3 case (of the order 100 nW or 500 µJ/cm^2^) and the *n* = 7 case (of the order of 1 nW or 5 µJ/cm^2^). We exclude excitation-induced thermal effects as no redshifts of the exciton resonances were detected in the experiments (for details see SI, Sec. 3).

**Fig. 3:**
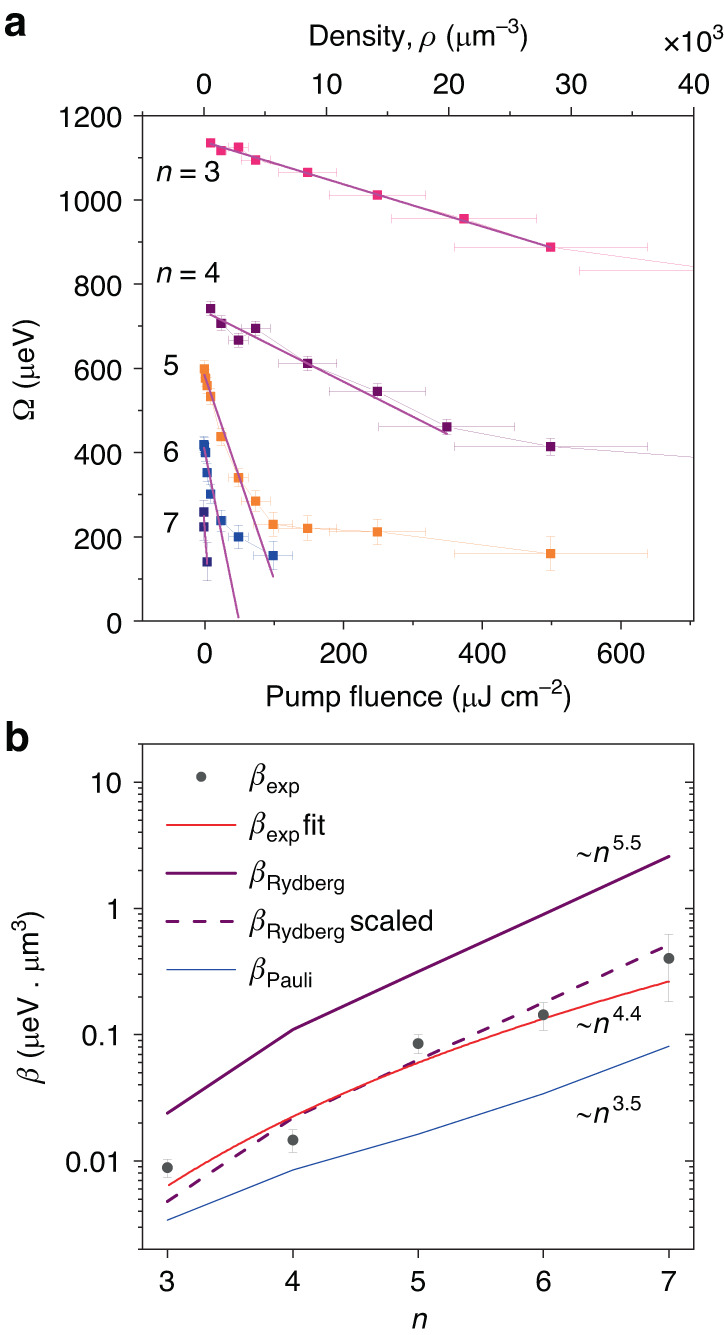
Nonlinear Rabi splitting decrease and its scaling. **a** Rabi splitting vs exciton density *ρ* and/or pump fluence is shown for different principal quantum numbers *n*, and reveals an *n*-dependent nonlinear coefficient. Data are extracted from spectra in Fig. 2. The lines show the linear trend seen at low density. **b** Linear-log plot of nonlinear coefficients (*β*-factors) vs principal quantum number *n*. Filled dots correspond to the experimental *β-*factor extracted from Fig. 3a, which can be fitted with an *n*^4.4±1.8^ dependence (solid red curve, error for 90% confidence level). The dashed purple and solid blue curves show the calculated *β*-factors for Rydberg and Pauli blockade, respectively. The solid purple curve depicts Rydberg saturation scaling for the full blockade (*C*_6_ ≈ 10^−15^ *n*^11^ meV µm^6^). (All calculated error bars are for standard error)

The fitting of the spectra at each power is performed using a coupled oscillators model previously used for Rydberg exciton-polaritons^33^, taking into account the spectral profile of the pulse and with the coupling strength being the main fitting parameter (see Methods and SI, Sec. 1). The model fits the experimental data well, in particular at low powers. From these fits we can extract the Rabi splitting Ω as a function of exciton density *ρ*, which is plotted in Fig. 3 (see Methods for the equation to deduce *ρ* and SI, Sec. 5 for its derivation). We stress that *ρ* being the excitonic fraction of resonantly pumped polaritons is an important parameter that defines the absolute value of nonlinear energy shifts. At low pump power, the overall Rabi splitting drops with quantum number *n*, since larger exciton size leads to smaller oscillator strength^35^. The Rabi splitting is also observed to decrease strongly with *ρ* or pumping power, showing a fast initial drop and overall nonlinear scaling with density at larger occupations. We also note that deducing this drop becomes difficult at larger *n* as the absolute value of the linear light-matter coupling decreases, while the cavity decay rate remains the same.

The exciton-polariton Kerr-like nonlinearity due to the reduction of Rabi splitting Ω with density *ρ* is usually characterised by the coefficient *β* = *d*Ω*/dρ*^36–38^. Classically, the Rabi splitting arises because the excitonic oscillators in the Cu_2_O active region add a frequency-dependent contribution to the refractive index which modifies the cavity resonance condition^39^. The magnitude of this refractive index component, as well as the Rabi splitting, decreases as *ρ* grows. Thus we can directly relate the nonlinear refractive index of Cu_2_O to *β* (see refs. ^36,40^ and references therein, and SI).

Here we focus on the nonlinear response at lower densities when the polariton doublets are still resolved and Ω behaves nearly linearly with *ρ*. The deduced *β* factors are plotted in Fig. 3b as a function of the principal quantum number *n* of polaritonic states (black dots, labelled as *β*_exp_). We observe rapid increase of nonlinearity as a function of *n* (note the log-linear scale), and the fitting provides scaling *β*_exp_ ∼*n*^4.4±1.8^ (red solid curve in Fig. 3b) (see SI, Sec. 7.1). The *β*-values are found to be between 0.01 µeV µm^3^ for *n* = 3, to 0.4 µeV µm^3^ for *n* = 7 (note, here we use volume units as natural for bulk crystal in a cavity). Already for *n* = 5 this nonlinearity exceeds that in other highly interacting polariton platforms, such as microcavities with GaAs-based quantum wells, if one takes into account excitonic spatial confinement (see SI, Sec. 6). Below we use the experimentally obtained *β* values and scaling to analyse main potential contributions and compare with our theoretical model.

### Theoretical analysis

To explain the experimental results we develop a theory for describing the effective decrease of light-matter coupling. Specifically, we take into account dipole–dipole interactions between Rydberg states of p-wave excitons, which are known to reduce Cu_2_O absorption with increasing exciton density in the cavity-free case^20^. This phenomenon may be explained as the formation of a blockade region in the vicinity of spatially extended exciton. In this region, light can no longer create new excitons due to the strong dipole–dipole interaction shifting the exciton energy out-of-resonance. Consequently, the optically active region in a sample decreases with the increase of exciton density.

We model this blockade effect and plot the *β*_Rydberg_ dependence using the theoretically predicted values of dipole–dipole interactions^41^ (solid purple curve in Fig. 3b). We observe that *β*_Rydberg_ scales as ∼ *n*^5.5^ (SI, Sec. 7.2). In this theoretical plot, a full blockade is assumed where the blockade region is defined sharply by a step-like boundary at the Rydberg blockade radius (*r*_*C*_). In reality, the blockade effect is coming from the exciton’s density-density correlations such that the transition between blockaded and non-blockaded regions is smooth^25^, meaning that the creation of additional exciton within *r*_*C*_ is not strictly forbidden. Therefore, the full blockade result gives approximately the upper bound which is likely to overestimate the nonlinearity. Furthermore, since the dipole–dipole interaction constants are generally difficult to calculate exactly and may be also overestimated^20,41^. To compare, we plot a scaled line (by a factor of 1/5) for the strength of dipole–dipole interaction, which matches the overall trend for experimental nonlinearity and provides a good fit (dashed purple curve in Fig. 3b).

Another possible origin of the reduction of Rabi splitting with increasing density is a nonlinear phase-space filling (NPSF) in polaritonic systems, as commonly observed with ground state s-excitons^36,38^. Nonlinear phase-space filling, also known as nonlinear saturation, is the statistical effect that emerges from the non-bosonic behaviour of excitons at large occupations. As excitons are composite quasiparticles, the Pauli blockade prevents the excitation of certain excitonic configurations if they are already occupied. This nonlinear decrease in the density of states leads to the effective reduction of light-matter coupling. Similarly to the Rydberg-induced case, the nonlinearity also grows with the exciton size, as a smaller number of excitons can be created (per volume or area) until the medium becomes effectively transparent. The blue curve in Fig. 3b shows the scaling of Pauli-induced nonlinearity for the experimentally deduced Bohr radius *a*_0_ = 0.83 nm (for *n* = 1, see SI, Sec. 7.2). The *β* factor associated with the Pauli blockade (*β*_Pauli_) has an asymptotic ∼*n*^2.5^ scaling, while at low *n* it is described by a ∼*n*^3.5^ dependence (SI, Sec. 7.2). The Pauli blockade curve, which has no fitting parameters, is well below the experimental values. To at least partially fit the experimental values using only the Pauli blockade, the Bohr radius must be set to *a*_0_ ≈ 2 nm. This greatly exceeds the exciton radius estimates from the measured low-density absorption (SI, Sec. 7). Therefore, we conclude that the Pauli blockade alone cannot explain the observed nonlinearity and that the Rydberg-induced blockade plays a dominant role. The contribution of the Rydberg blockade to exciton-polariton nonlinearity is an order of magnitude stronger (see Fig. 3b).

## Nonlinear *n*_2_ parameter

**Fig. 4:**
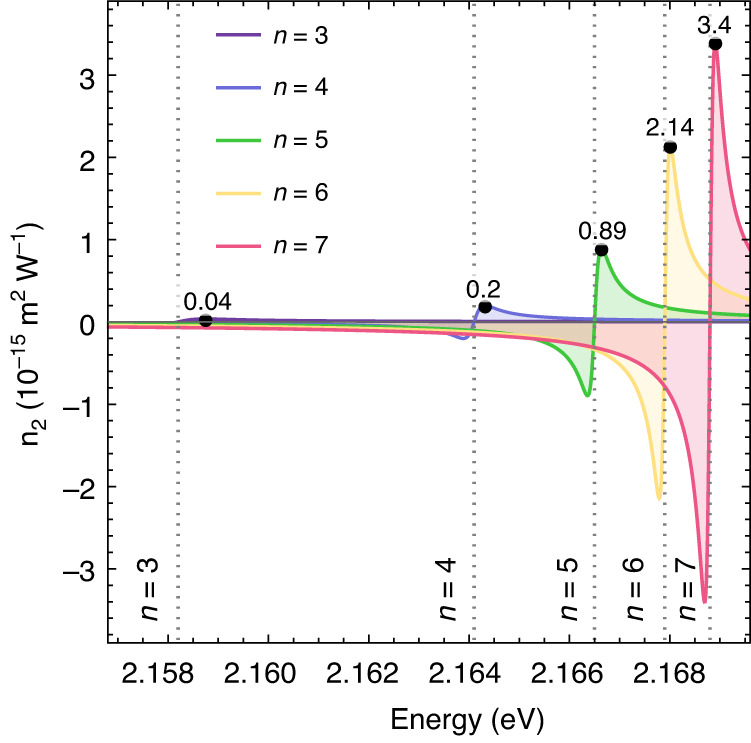
Nonlinear refractive index *n*_2_. Coloured curves show contributions from each excitonic mode with *n* = 3*,…*,7. Here we use *β* = 1.41 × 10^−5^*n*^5.5^ µeV µm^3^ corresponding to the dashed purple curve in Fig. 3

From the nonlinear optics perspective, the polariton nonlinearity can be also characterised by the nonlinear refractive index *n*_2_ of the active medium in a microcavity (Cu_2_O in our case)^36^. This nonlinear parameter appears in the total refractive index as a frequency- and intensity-dependent term, *n*_T_(*ω*) = *n*_0_(*ω*) + *n*_2_(*ω*)*I*. The *n*_2_ parameter from blockade effects may be estimated by using Eq. (1) (see SI, Sec 7.3):

1 $${{{n}}}_{2}\left({{\omega }}\right)\approx -\frac{{h}_{{{n}}}{\beta }_{n}}{2c{{{n}}}_{0}^{2}\omega }\frac{{G}_{n}^{(0)}\left(\omega -{\omega }_{n}\right)}{{\left(\omega -{\omega }_{n}\right)}^{2}+{\frac{1}{4}\gamma }_{n}^{2}}$$

where ${G}_{n}^{\left(0\right)}$ is the light-matter coupling constant, *ω*_*n*_ is the Rydberg resonance frequency, *γ*_*n*_ is the excitonic linewidth, *n*_0_ is the background refractive index of Cu_2_O, *c* is the speed of light in vacuum and *h*_*n*_ is a constant of proportionality (determined from the measured Rabi splitting using Eq. (S25), see SI, Sec. 7.3). Using the *β*-factors in Fig. 3b, we derive the energy dependencies of *n*_2_ for each individual excitonic mode in Fig. 4. Black dots show the upper bounds of *n*_2_ obtained using the theoretical estimates of *β*, with values ranging from 10^−17^ to 10^−15^ m^2^/W, for *n* = 3 to *n* = 7. Using the experimentally measured *β*-factors we deduce peak *n*_2_ values ranging from 10^−17^ m^2^/W to 4 × 10^−15^ m^2^/W, for *n* = 3 to *n* = 7. The peak values measured for *n* = 7 exciton resonance are comparable to *n*_2_ in GaAs polariton waveguides^40^.

These values of *n*_2_ are 8 orders of magnitudes lower than those measured on bulk Cu_2_O in ref. ^21^, where CW excitation was used in resonance with Rydberg excitons. However, we note that studies of polariton nonlinearity in GaAs-based photonic systems showed that CW excitation usually results in observed effective *n*_2_ values one to two orders of magnitude bigger than in the case of picosecond pulsed pumping. Such a discrepancy was explained by a population of long-lived (up to 100 ps) excitons by the CW beam, the interaction with which leads to enhanced nonlinear energy shift^42^. In the case of Cu_2_O, the CW excitation in the vicinity of Rydberg exciton resonances and the subsequent relaxation of the photoexcited carriers to the ground 1 s level is expected to lead to high population of long-lived (with a lifetime of 13 µs^43^) dark 1 s paraexcitons. Lifetimes up to several hundred microseconds were also reported, which was attributed to long-lived excitons trapped at defects or unknown metallic impurities^44^. At sufficiently high density long-lived 1 s excitons or excitons trapped to defects may further recombine through Auger recombination creating plasma. The exact density of 1 s excitons and plasma in this case may depend on the particular sample and the number of defects at which 1 s excitons may accumulate.

A possible explanation for the very high values of *n*_2_ observed in ref. ^21^ is therefore an interplay between resonantly pumped Rydberg excitons and plasma. Free carriers may increase the strength of the Rydberg blockade through screening, which increases the size of the Rydberg excitons leading to enhancement of the dipole–dipole interactions^24^. At the same time, the screening may reduce the oscillator strength of the excitons resulting in a density-dependent change in their contribution to the refractive index. In support of this suggestion, we note that Heckötter et al.^45^ characterised the influence of free carriers, showing that absorption for *n* = 10 Rydberg excitons reduces by a factor of ∼3 at free carrier density as low as ∼0.5 µm^−3^. In the following section, we use time-resolved measurements to shed some light on the regimes where the polariton nonlinearity is dominated by ultra-fast processes, such as Rydberg blockade, and where it may be complicated by slower processes such as Auger-mediated generation of plasma.

## Pump-probe experiment

Importantly, in the single pulse experiment presented above the polariton nonlinearity that we measured must be ultra-fast since it develops within the picosecond timescales of the probe pulse (which is also the pump pulse in that case) and the polariton lifetime. However, some of the resonantly excited exciton-polaritons can be absorbed, with the resultant formation of lower energy excitons, and then plasma through the Auger exciton recombination. Therefore the Rabi splitting may remain quenched for some time after the pump pulse is gone due to interaction with this long-lived plasma. To reveal this effect we further perform pump-probe measurement of Rabi splitting for the *n* = 4 resonance. The *n* = 4 polaritons were excited with a strong pump and the transmission of the sample was measured using a much weaker probe pulse delayed by some time from the pump. The linear polarisation of the probe is chosen to be perpendicular to the pump and the transmitted pump beam was rejected by a linear polariser. The *n* = 4 state was chosen because it provides a good signal-to-noise ratio for small probe powers.

**Fig. 5:**
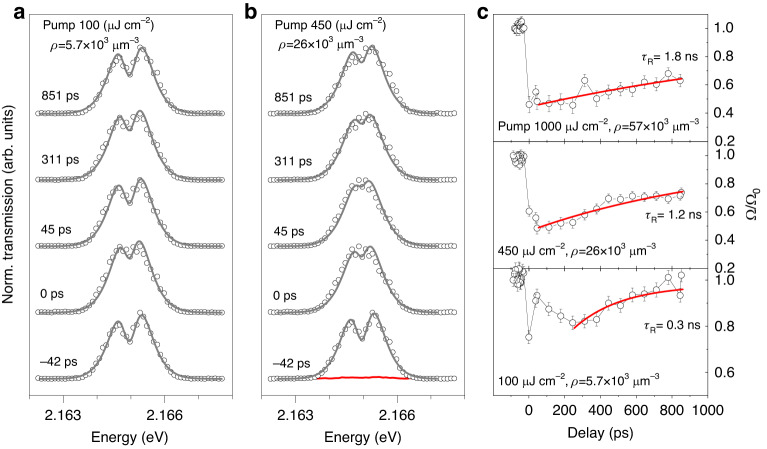
Pump-probe of the Rabi splitting at *n* = 4. **a** Normalised transmission spectra at different delay times between pump and probe pulses at fixed pump(probe) fluence 100 µJ cm^−2^ (50 µJ cm^−2^). Solid grey lines correspond to fits. **b** Normalised transmission spectra at different delay times between pump and probe pulses at fixed pump(probe) fluence 450 µJ cm^−2^ (50 µJ cm^−2^). The red line corresponds to pump only transmission signal (≈30 times smaller than the probe after polarisation rejection). Solid grey lines correspond to fits. **c** Rabi splitting normalised by Rabi splitting at negative time delays (Ω*/*Ω_0_) extracted from the spectra and plotted as a function of delay time between pump and probe at three pump fluences (100, 450 and 1000 µJ cm^−2^). Solid red lines are single exponent fits

We plot the results of the pump-probe experiment in Fig. 5. In Fig. 5a, b we show the transmission spectra for selected delays between pump and probe pulses for pump fluences of 100 µJ cm^−2^ and 450 µJ cm^−2^, respectively. The probe fluence was 50 µJ cm^−2^. The transmission spectra are then fitted to extract the Rabi splittings as a function of the time delay. In this fitting procedure, we omit those time delays between −29 and 37 ps (apart from zero delay), for which the interference between the residual pump and probe prevents reliable fitting (the residual pump intensity in the polarisation of the probe is about 30 times less than that of the probe for pump fluence 450 µJ cm^−2^ and can not be suppressed completely by the linear polariser, see SI, Sec. 9).

At pump fluence of 100 µJ cm^−2^ the Rabi splitting is reduced at *t* = 0 as expected for the instantaneous Rydberg exciton-polariton blockade mechanism and by *t* ≈ 40 ps has recovered almost back to its value prior to the pump arrival (Fig. 5c, bottom panel). At longer delay times *t* > 40 ps the Rabi splitting reduces and then increases again on a timescale of 200–300 ps. We attribute this behaviour to the 1 s exciton and plasma dynamics created by the pump pulse, which is expected to occur on a nanosecond timescale at exciton densities 10^3^–10^4^ µm^−3^ given the Auger recombination rate of 1 s excitons of the order of 10^−4^ µm^3^/ns^46^. At higher pump fluences of 450 µJ cm^−2^ and 1000 µJ cm^−2^, shown in the middle and upper panels of Fig. 5c, the creation of plasma after the pump pulse is expected to occur on a shorter timescale and indeed we observe the Rabi splitting is rapidly quenched and then recovers monotonically on timescales of 1.2 and 1.8 ns, respectively. The exact explanation of the observed temporal dynamics requires complex modelling of the exciton-plasma conversion and decay using the corresponding rate equations^46^ and is beyond the scope of this manuscript.

Finally, we also study Rydberg exciton-polariton transmission spectra under non-resonant CW excitation well above the band gap of the Cu_2_O. In this case, we find that the collapse of the Rabi-splitting is observed at photon densities about 6 orders of magnitude less than in the pulsed excitation regime (see SI, Sec. 8). This experiment further confirms the important role of long-lived 1 s states and plasma, the density of which can be significantly higher in CW than pulsed excitation.

## Conclusion

In conclusion, for the first time, we investigated the nonlinear behaviour of a polaritonic system based on Cu_2_O in the ultra-fast pulsed regime. The polariton system allows us to probe the collapse of the Rabi splitting for Rydberg exciton-polaritons and we observed that the associated polariton nonlinearity increases as ∼*n*^4.4±1.8^ with principal quantum number *n* due to stronger dipolar exciton–exciton interactions. The experimental values of polariton nonlinearity coefficient *β* are in good agreement (within a factor of 5) with our microscopical model, which takes into account both the Rydberg dipole–dipole interactions and Pauli blockade without fitting parameters. Furthermore, our pump-probe data suggests that there are several contributions to the nonlinearity in Cu2O-based systems, which act on several different timescales. As well as the ultra-fast response there are contributions which can persist significantly longer than pulse duration and polariton lifetime. The timescales for these are consistent with the population of long-lived states and the creation of plasma. In the CW excitation regime, these can be even more pronounced resulting in greatly enhanced nonlinearity. In order to investigate the effect of plasma on Rydberg exciton or exciton-polariton blockade in more detail, and to fully explain the drastic differences observed in nonlinearities for pulsed and CW excitation, we suggest further studies using for example pulsed excitation with varying pulse duration and repetition rate.

We note that higher exciton-polariton nonlinearities are possible to achieve by modification of several factors: (i) use higher quality crystals and observe higher *n* states, (ii) use higher quality microcavities to increase strong coupling with high *n* states, (iii) reduce the thickness of Cu_2_O to the quantum well level to decrease absorption losses in the cavity and enhance exciton–exciton interactions, and (iv) exploit electromagnetically induced transparency for the reduction of losses due to phonons^47^. Our work demonstrates that Rydberg polaritons in Cu_2_O are a suitable platform for quantum polaritonics with nonlinearities that scale sufficiently strongly with Rydberg exciton quantum number to reach the single polariton nonlinearity.

## Methods

### Sample and setup

Our cavity containing a natural Cu_2_O sample is cooled down to 4 K in a continuous flow liquid Helium cryostat. Natural Cu_2_O crystals are employed here since these are of higher quality than artificially grown samples^19,48^. To prepare the microcavity sample, the Cu_2_O flake was first cleaned in xylene with 1 min of delicate sonication and rinsed in isopropanol. Producing the mirror layers was done by initially securing the Cu_2_O flakes onto a substrate with a 45 mg/ml solution of PMMA and toluene. The sample with the Cu_2_O flake was then loaded into an ˚Angstrom Engineering thermal evaporator. Silver was then evaporated onto the Cu_2_O using a resistive source at a rate of 0.2 nm *s*^−1^. The final thickness of the silver mirror was 50 nm. The sample was then unloaded and the Cu_2_O flake was removed and carefully rotated to expose the opposite side that has no silver deposited. The PMMA solution was then used to secure this to a new substrate and the deposition was repeated with the same thickness. To attach the resulting structure on the final substrate, PMMA 495 resist in 8% anisole was spun at 4000 rpm on a glass slide resulting in a 600 nm thick layer. The flake was transferred onto the resist and then baked for 5 min at 180 °C.

The Fourier space imaging spectra in Fig. 1 have been obtained with a 1 ns super-continuum laser with a repetition rate of 23 kHz and a spectral width after filtering of 50 nm. In order to achieve narrowband resonant excitation in Fig. 2, we used 100 fs pulses at 1 kHz repetition rate obtained from the frequency-doubled output of a TOPAS optical parametric amplifier and then filtered by a 4f configuration pulse shaper with a 1200 g/mm grating and a slit slightly displaced from the focal plane to obtain a Gaussian shape spectrum with FWHM 1.75 meV.

### Fitting procedure and extraction of β factors

The transmission spectra resulting from resonant excitation consist of a doublet centred around the excitonic resonance. In order to extract the coupling strength of the Rydberg polariton from the transmission spectra, we fit the data according to the model used in^33^ taking also into account the small spectral width (FWHM = 1.75 meV) of the excitation pulse [see SI, Sec. 1, Eq. (S1)]. Cavity and excitons linewidths in the fit were fixed for all powers and obtained in separate measurements for each *n* (see SI, Sec. 2). Two fitting parameters were allowed to vary with power: the amplitude and the coupling strength. Thus we extract the coupling strength dependence on average power. To extract *β* we plot the Rabi splitting as a function of resonantly excited exciton density estimated from the transmitted power through the cavity in resonant conditions and fit it with the linear function. The slope provides an average estimate for *β*.

The density of excitons *ρ* per volume is calculated from the incident average excitation power *P*_avg_ by using the following equation:

2 $$\rho =\frac{{T}{P}_{{{\rm{avg}}}}{\tau }^{{\prime} }{\left|X\right|}^{2}}{f{\tau }_{p}{LA}{{\hslash }}\omega {\left|C\right|}^{2}}$$

where *T* = 1*/*180 is the fraction of incident power that is transmitted, *τ*^′^ = 14 ps is the inverse of the tunnelling rate of photons out of the cavity, through the silver mirror and towards the detector, *f* = 1 kHz is the laser repetition rate, *τ*_p_ ≈ 1.57 ps is the effective laser pulse width, *A* ≈ 20 µm^2^ is the effective area for the interaction^28^, *L* = 26 µm is the cavity length, ℏ*ω* is the single-photon energy and |*C*|^2^ = 0.5 and |*X*|^2^ = 0.5 are photonic and excitonic fractions of the polaritons respectively (see SI, Sec. 5 for more details).

### Supplementary information

Supplementary Information

## Data Availability

The data supporting these findings are freely available from the corresponding author upon reasonable request.

## Code availability

The code used for analysis is freely available from the corresponding author upon reasonable request.
