# Supplementary material for: Nonlinear Rydberg exciton-polaritons in Cu2O microcavities
Source: Light Sci Appl. 2024 Feb 6;13:47. doi: 10.1038/s41377-024-01382-9 (PMC10847413; doi:10.1038/s41377-024-01382-9)
Supplement: Supplementary file 1 — Supplementary Information [file 41377_2024_1382_MOESM1_ESM.pdf]

Nonlinear Rydberg exciton-polaritons in Cu_2O microcavities

Maxim Makhonin[1], Anthonin Delphan[1], Kok Wee Song[2], Paul Walker[1], Tommi Isoniemi[1], Peter Claronino[1], Konstantinos Orfanakis[3], Sai Kiran Rajendran[3], Hamid Ohadi[3], Julian Heckötter[4], Marc Assmann[4], Manfred Bayer[4], Alexander Tartakovskii[1], Maurice Skolnick[1], Oleksandr Kyriienko[2] and Dmitry Krizhanovskii[1]

[1]Department of Physics and Astronomy, University of Sheffield, Sheffield, S3 7RH, United Kingdom.
[2]Department of Physics and Astronomy, University of Exeter, Stocker Rd, Exeter, EX4 4PY, United Kingdom.
[3]School of Physics & Astronomy, University of St Andrews, St Andrews, KY16 9AJ, United Kingdom.
[4]Fakultät Physik, TU Dortmund, August-Schmidt-Straße 4, Dortmund, 44227, Germany.

Supplementary Information

1 Transmission spectra fitting procedure **3**

2 Measurement of excitonic and cavity linewidths **3**

3 The effect of temperature on the exciton resonances **5**

4 Strong coupling in broadband excitation regime up to $n = 7$ **6**

5 Calculation of the density **7**

6 Comparison of nonlinearities **10**

7 Theoretical analysis **10**
7.1 Rabi splitting and light-matter coupling 11
7.2 Rydberg and Pauli blockade 12
7.3 n_2 parameter from Rabi frequency measurement 15

8 Non-resonant pumping and quenching of Rabi Splitting **16**

9 Pump-probe zero delay point **18**

1 Transmission spectra fitting procedure

To fit the transmission spectra we used the model of cavity transmission from Ref. [1], multiplied by a Gaussian function which accounts for the spectrum of the pulse incident on the cavity. This reads

$$T_n \approx \frac{A \exp\left(-2\left(\frac{\Delta_n}{\sigma}\right)^2\right)}{\left(\frac{\kappa}{2} + G_n^2 \frac{\gamma_n/2 + 2Q_n\Delta_n}{\gamma_n^2/4 + \Delta_n^2}\right)^2 + \left(\Delta_n - G_n^2 \frac{\Delta_n - Q_n\gamma_n}{\gamma_n^2/4 + \Delta_n^2}\right)^2}, \tag{S1}$$

where A is the peak amplitude, Δ_n is the laser frequency detuning from the excitonic resonance with principal quantum number n, γ_n is the excitonic linewidth for corresponding n, κ is the cavity linewidth, G_n is the coupling strength, Q_n is the Fano asymmetry parameter, and σ is the pulse spectral width. In all cases the subscript n refers to the exciton with index n. We use Eq. (S1) to fit the transmission spectra at each n by fixing the parameters γ_n, κ and Q_n corresponding to each excitonic resonance taken from separate measurements (see Section 2 below). For each excitonic resonance we perform a global fit over all excitation powers, with only the coupling strength G_n and the amplitude A varying as a function of power. Thus we obtain power dependence of G_n.

2 Measurement of excitonic and cavity linewidths

In Fig. **S1**, we extract the photonic cavity linewidth κ of several modes at different energies. The data are taken from angle-resolved transmission (also called Fourier imaging or $\boldsymbol{k}$-space imaging) of a broadband super-continuum laser. A section of the angle-resolved data at zero incidence angle to the sample normal ($k = 0$) is presented in the figure. As we want to extract an uncoupled and unperturbed "purely photonic" cavity mode, the spectra are taken from a spatial region of the sample where the cavity modes are detuned far away from the excitonic resonances. This occurs due to a small wedge in the thickness of the sample.

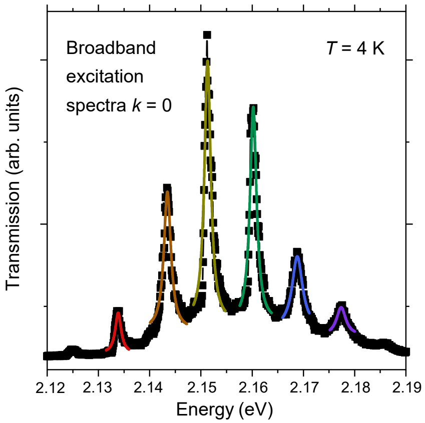

Fig. S1: Normal incidence transmission spectrum of a Cu_2O microcavity under broadband excitation, showing uncoupled Fabry–Pérot cavity modes. Points are experimental data. Solid curves show Lorentzian lineshape fits to the modes.

From the Lorentzian fits in Fig. S1, we can extract the cavity linewidth as a function of energy, which we plot in Fig. S2. The trend can then be extrapolated to find appropriate κ values for energy levels matching exciton resonances.

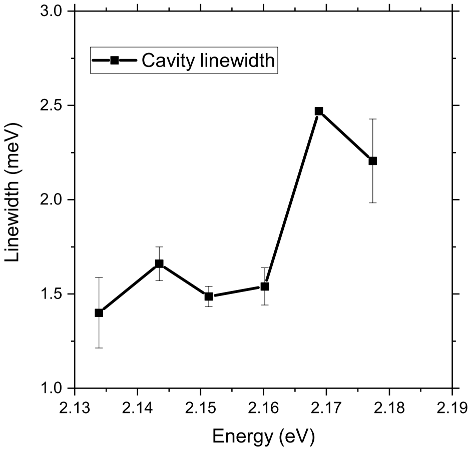

Fig. S2: Cavity linewidth extracted from Fig. S1 as a function of energy.

From white light transmission spectra of a bare Cu_2O flake with excitonic resonances fitted as an asymmetrical Fano resonances we extract exciton linewidth γ_n and Fano asymmetry parameter Q_n (see Fig. S3).

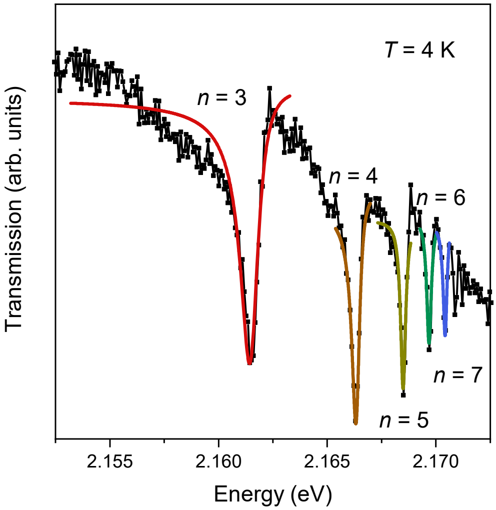

Fig. S3: Transmission spectra of a bare Cu_2O flake. Points are experimental data. Solid curves are Fano lineshape fits for each resonance.

3 The effect of temperature on the exciton resonances

We believe the laser induced heating effects are negligible in our experiment. To verify this we performed an additional measurement where the exciton resonances were monitored in transmission in a bare Cu_2O crystal using white light for different temperatures. With the increase of temperature of the sample from 4 to 20 K we observed a significant red shift of the exciton resonances by about 0.6 meV as shown in the Fig. S4, whereas the exciton linewidth and the dip of the exciton resonance stay almost the same, indicating no reduction of the exciton oscillator strength with temperature. By contrast, our measurements of the polariton resonances for different powers in Fig. 2 of the main text do not show any red shift of the bare exciton resonances within the spectrometer resolution ~ 0.1 meV: the energy positions of the dip between the polariton resonances corresponding to the exciton levels do not change. This indicates that the sample temperature does not change with increase of pump power and the reduction of the exciton-photon coupling can not be explained by heating.

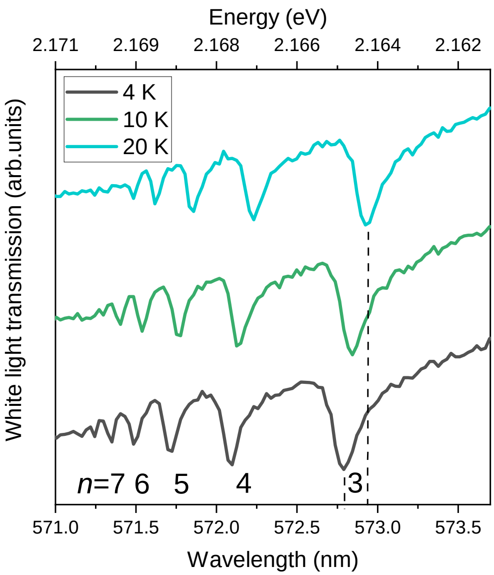

Fig. S4: White light transmission spectra of a bare Cu_2O flake at different temperatures.

4 Strong coupling in broadband excitation regime up to $n = 7$

In Fig. 1 of the main text we demonstrated strong coupling in the broadband excitation regime using angle-resolved transmission spectra, by showing the anti-crossing of the cavity modes around the excitonic lines for $n = 3$, $n = 4$, and $n = 5$. In this section, we show that such anti-crossing can be observed for up to $n = 7$ in the broadband excitation regime, similarly to the narrowband excitation scheme demonstrated in the main text of the article.

While Fig. 1 of the main text was obtained using $\boldsymbol{k}$-space imaging, it was not possible to use this technique for higher n. Indeed, the cavity modes become noisy and harder to resolve at higher energy, which makes the identification of such modes challenging. Instead, the anti-crossing is obtained by scanning the excitation position on the sample, which results in the cavity modes shifting due to the slight wedge in sample thickness and thus changing their detuning with respect to the excitons. This is similar to the technique used in Ref. [1]. When the modes cross the exciton resonance, a doublet and splitting characteristic of strong coupling are observed. This scan across different positions on the sample is shown in Fig. S5.

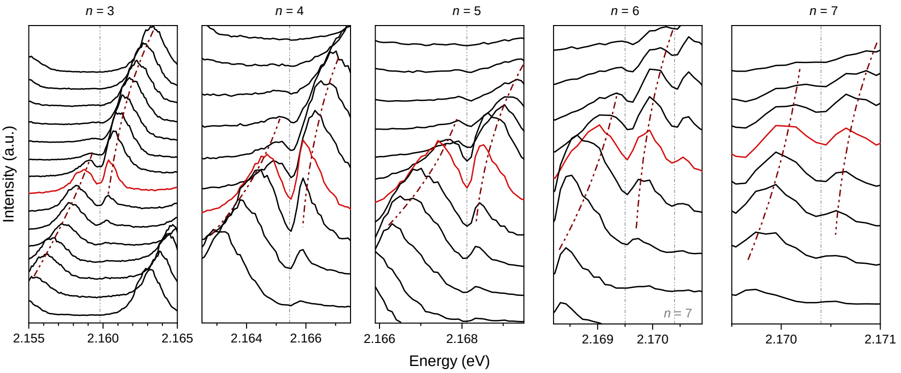

Fig. S5: Broadband excitation and position scan across the sample showing anticrossing for excitonic resonances from $n = 3$ to $n = 7$. Brown dash-dot lines are a guide to the eye showing positions of upper and lower polariton modes. Grey lines are showing positions of exciton resonances.

5 Calculation of the density

The density of photons inside a cavity can be calculated from the power emitted by the cavity together with the rate of escape through the mirrors, which have a finite transmission coefficient. A detailed explanation of the principle can be found in the supplementary information of Ref. [2]. In the present work, the power emitted by the cavity was measured directly, while the transmission of the mirrors was calculated using a transfer matrix model of the sample with calibrated parameters. Transfer matrix modelling is a standard technique which exactly solves Maxwell's equations for planar layer structures of the type we use in this work. In our work we deduce the size of the nonlinearity at the lowest powers, in the limit where the interaction energy compared to the the other energy scales in the system, such as losses, are tending towards zero. We therefore use the reasonable approximation that in this limit the density of particles in the cavity can be deduced using a linear model of the cavity electromagnetic response.

The first step in the model was to calibrate the reflection, transmission and absorption of the silver mirrors. In the same deposition runs in which the mirrors were deposited onto the Cu_2O to form the cavity the same silver films were also deposited onto a glass substrate. We then measured the transmission and reflection of these silver films using the same laser as in the main experiments, and a commercial laser power meter. We measured reflection of $R = 92 \pm 3\%$ and transmission of $T = 2.0 \pm 0.3\%$. The silver thickness of 51.5 nm was known from the deposition rate and time. We then used the transfer matrix method to model the reflection and transmission of the structure and found the silver refractive index that gave the measured transmission and reflection. We find real and imaginary parts of the refractive index n $= 0.24 \pm 0.11$ and $k = 3.78 \pm 0.10$ respectively. Here the errors were

obtained by propagating the errors in R and T using the numerically calculated gradients $\partial \mathrm{n}/\partial T$, $\partial \mathrm{n}/\partial R$, $\partial k/\partial T$, and $\partial k/\partial R$. For the error $\Delta \mathrm{n}$ in n we used the formula $\Delta \mathrm{n} = |\partial \mathrm{n}/\partial T|\Delta T + |\partial \mathrm{n}/\partial R|\Delta R$, and similar for the error Δk in k. We note that our refractive index is also consistent with values from the literature [3]. We checked that our deduced values of density do not vary by more than a few percent with the real part of the refractive index of the silver mirrors.

Next we calibrated the Cu_2O layer properties. The background refractive index 2.7386 was taken from the literature [4]. We then modelled transmission through the cavity using the transfer matrix method and varied the thickness of the Cu_2O until the free spectral range between adjacent photonic modes matched that in the experiment. In this manner we found a cuprous oxide thickness of 26.0 micrometers. The imaginary part of the cuprous oxide refractive index, 0.000483, was then found so that the quality factor of the calculated modes was 2000 away from the excitonic resonances, in agreement with the experimental results. We checked that our deduced values of density do not depend (by more than a few percent) on the refractive index of the cuprous oxide provided that the free spectral range matched the experimental value.

Having calibrated both mirror and cavity properties we then used the transfer matrix model to calculate both the energy density inside the cavity and the Poynting flux (power per unit area) outside the cavity for a monochromatic incident laser, as in Ref. [2]. Integrating the energy density over the cavity length and dividing by the flux gives τ', the inverse of the rate of tunnelling of photons through the mirror. We then have $P_{\mathrm{out}} = E_{\mathrm{cav}}/\tau'$ where P_{out} is the measured power coming out of the cavity and E_{cav} is the energy stored as photons inside the cavity.

We calculated τ' as a function of photon wavelength and found variation of only 0.4% over the 1.75 meV (0.467 nm) energy range corresponding to the bandwidth of the spectra shown in the experiments. Since in the linear regime the time-varying fields inside the cavity may be viewed as a superposition of different frequency waves (by the Fourier transform) we can therefore assume that the ratio of stored energy to output power is the same for the pulsed case as for the monochromatic case. We arrive at $\tau' = 14 \pm 2$ ps. Here the uncertainty $\Delta\tau'$ in τ' was obtained by propagating the errors $\Delta \mathrm{n}$ and Δk in the silver mirror real and imaginary refractive indices respectively using $\Delta\tau' = |\partial\tau'/\partial \mathrm{n}|\Delta \mathrm{n} + |\partial\tau'/\partial k|\Delta k$. The gradients $\partial\tau'/\partial \mathrm{n} = 3.27$ ps and $\partial\tau^{'}/\partial k = 19.48$ ps were obtained numerically by running the transfer matrix simulation over a range of different refractive indices.

The number of particles in the cavity is related to the energy by $N = E_{\mathrm{cav}}/\left(\hbar\omega\right)$ where ω is the central angular frequency of the pulses. The areal density of photons $\rho_{\mathrm{photons}} = N/A$ can then be obtained using the effective area A for the nonlinear interaction. This is given by [5] $1/A = \int\int_{-\infty}^{\infty} I^2(x,y)dxdy$ where $I(x,y)$ is the normalised spatial intensity distribution inside the cavity, which in our case has a Gaussian shape. This takes account of the fact that the

density varies over the Gaussian spot and so we measure a weighted average of more and less strongly interacting regions.

In a similar way our time-averaged measurement of the $\sim$ 1 ps pulses coming from the cavity is an average over the time-varying interaction energy in the cavity weighted by the occupancy of the cavity. Both the interaction energy and occupancy are proportional to the temporal shape of the cavity occupancy $I(t)$. Taking this weighted average gives the effective pulse length τ_{p} using $1/\tau_p = \int_{-\infty}^{\infty} I^2(t)dt$. Transform limited Gaussian pulses with 1.75 meV spectral width have a temporal width of 1.0 ps. This is then a lower bound for the possible temporal width of the density in the cavity, which provides an upper bound for the density and hence a lower bound for the nonlinearity. However, the real temporal width is likely to be longer due to partial cavity filtering of the incident spectrum and/or chirp in the incident pulses. Using the lower bound of a 1 ps Gaussian we obtain $\tau_{\mathrm{p}} = 1.57$ ps.

We then use $E_{\mathrm{cav}} = P_{\mathrm{out}}\tau'$ from above and insert the effective output pulse power $P_{\mathrm{out}} = P_{\mathrm{avg,T}}/\left(f\tau_{\mathrm{p}}\right)$. Here $P_{\mathrm{avg,T}}$ is the average power of the *transmitted* beam, $f = 1$ kHz is the laser repetition rate and τ_{p} is the effective pulse length defined above. We finally make the substitution $P_{\mathrm{avg,T}} = TP_{\mathrm{avg}}$ where P_{avg} is the average *incident* beam power and $T = 1/180$ is the measured transmission through the cavity. This accounts for the reflection of spectral components of the incident pulse which are not resonant with the cavity modes. Recalling that $\rho_{\mathrm{photons}} = N/A = E_{\mathrm{cav}}/\left(A\hbar\omega\right)$ finally leads to an expression for the photon density in cavity:

$$\rho_{\mathrm{photons}} = \frac{TP_{\mathrm{avg}}\tau'}{f\tau_{\mathrm{p}}A\hbar\omega}. \tag{S2}$$

So far our discussion has concerned a purely photonic cavity with no strong exciton-photon coupling. When strong coupling is included the fundamental eigenstates of the system, the polaritons, are part photon and part exciton. The fractions of photon and exciton content are $|C|^2$ and $|X|^2$ respectively with $|C|^2 + |X|^2 = 1$. Since our measurements of nonlinearity are made at zero exciton-photon detuning we have $|C|^2 = |X|^2 = 0.5$. Only the photonic component of the polaritons leads to tunnelling through the mirror into free space modes outside the cavity. Thus Eqn. S2 still holds and the output power from the cavity gives the density of photons inside the cavity, where we are careful to remember that this is really the density of the photonic component of the polaritons. The density of the photonic component is related to the density of polaritons by $\rho_{\mathrm{photons}} = \rho_{\mathrm{polaritons}}|C|^2$. We can then write the density of polaritons as

$$\rho_{\mathrm{polariton}} = \frac{TP_{\mathrm{avg}}\tau'}{f\tau_{\mathrm{p}}A\hbar\omega|C|^2}. \tag{S3}$$

Finally, to obtain the density of excitons (the excitonic component of the polaritons) we multiply the polariton density by the excitonic fraction,

$$\rho = \frac{TP_{\mathrm{avg}}\tau'|X|^2}{f\tau_{\mathrm{p}}A\hbar\omega|C|^2}. \tag{S4}$$

6 Comparison of nonlinearities

We find that in our system the nonlinearity coefficient β, relevant for applications, ranges between 0.01 μeV μm^3 for $n = 3$, to 0.4 μeV μm^3 for $n = 7$ Rydberg exciton-polaritons (see Fig. 3 in the main text). It is important to note that in a Cu_2O cavity the excitons are delocalised within the cavity thickness of 26 μm, and so the exciton density is expressed per unit volume of the cavity region and the appropriate units for the nonlinear parameter are energy shift divided by number of particles per unit volume, that is μeV μm^3.

In other highly nonlinear polariton systems, such as for example microcavities with embedded (In)GaAs quantum wells, the excitons are confined within the thickness of the quantum wells (typically $\sim$ 10 nm per quantum well in the device). A single quantum well thickness is comparable to the exciton Bohr radius and hence the density is usually expressed per unit area of a single quantum well. For GaAs polaritonic systems the reported strengths of exciton-polariton nonlinearity (either β-values or g-values characterising the collapse of strong exciton-photon coupling or the exciton energy shifts, respectively) are in the range from 2 to 10 μeV μm^2 [2, 6–9]. In order to compare these to β-values of the bulk excitons we study, one has to transform the 2D density to effective 3D density by dividing it by the thickness of the quantum wells. This enables a unified characterisation of the strength of interactions between two excitons separated by a certain distance irrespective of how they are positioned within the cavity region, whether they are bulk or confined to a single or multiple 2D layers.

Following this procedure the 2D values of 2–10 μeV μm^2 in GaAs-based systems are equivalent to 0.02–0.1 μeV μm^3. These values are exceeded by the β values in Cu_2O microcavity already for $n = 5$ exciton-polaritons. Qualitatively, this is expected since the exciton Bohr radius for $n = 5$ is already of the order of 30 nm, being three times larger than that in GaAs, leading to stronger dipole-dipole interactions or Pauli blockade mechanism. Similarly, nonlinearities in hybrid perovskites containing order 3000 layers have been studied [10]. Each layer is of order 1.7 nm thick. The per-layer nonlinearity of 3 μeV μm^2 is equivalent to a bulk-like nonlinearity of 0.005 μeV μm^3. This 3D value is then convenient to deduce the effective 2D nonlinearity of perovskite structures with different numbers of layers.

7 Theoretical analysis

In this section we present the theoretical analysis of Rydberg excitons coupled to photons in a microcavity. In the first part, we focus on the relation of the Rabi splitting (Ω_n) and the light-matter coupling constant (G_n). In particular, we derive $\Omega_n \approx \Omega_n^{(0)} - \beta_n \rho$, where ρ is the exciton density, and $\Omega_n^{(0)}$ is the Rabi splitting at vanishing density, $\rho = 0$. This gives the theoretical beta factor β_n used to characterise the strength of nonlinearity. In general, $\beta_n \sim V_\mathrm{B}/V$ is mostly determined by the ratio between blockade (V_B) and the total volume (V). In the next part, we discuss the blockade V_B due to Rydberg and Pauli

blockade. These two different mechanisms lead to distinct scaling behavior. Also, we discuss the estimates for β_n in Cu_2O (Fig. **3b** in the main text). In the final part, we deduce the nonlinear refractive index n_2 for Cu_2O using the nonlinear polaritonic response.

7.1 Rabi splitting and light-matter coupling

Transmission of the Cu_2O microcavity system around n-th excitonic state can be modeled as [1]

$$T_n \approx \frac{1}{(\frac{\kappa}{2} + G_n^2 \frac{\gamma_n/2 + 2Q_n\Delta_n}{\gamma_n^2/4 + \Delta_n^2})^2 + (\Delta_n - G_n^2 \frac{\Delta_n - Q_n\gamma_n}{\gamma_n^2/4+\Delta_n^2})^2}, \tag{S5}$$

where κ is the cavity line-width, G_n is the exciton-photon coupling constant, Δ_n is the detuning, γ_n is the excitonic linewidth, and Q_n is the Fano asymmetry parameter. In the weak light-matter coupling regime ($G_n \ll \gamma_n$), the system only responds to light with frequency near the exciton resonance ($\Delta_n = 0$). One can see from Eq. (S5) that in the strong coupling regime ($G_n \gg \gamma_n$) the optical response changes qualitatively [11]. Namely, in this regime the resonance changes from $\Delta_n = 0$ to two resonances at $\Delta_n = \pm\frac{1}{2}\Omega_n$. This comes from the hybridisation of photonic and excitonic modes. The resulting states – polaritons – are quasiparticles which energies are characterised by the Rabi splitting Ω_n.

The Rabi splitting can be analytically calculated from Eq. (S5) by identifying the separation between points of maximum response in the transmission spectrum. For instance, in the absence of asymmetry ($Q_n = 0$), Rabi splitting can be obtained from Eq. (S5) as [12]

$$\Omega_n = 2\sqrt{G_n\sqrt{\tfrac{1}{2}\gamma_n(\kappa+\gamma_n) + G_n^2} - \tfrac{1}{4}\gamma_n^2}. \tag{S6}$$

We can see that the Rabi splitting explicitly depends on the coupling constants G_n and the linewidth γ_n. These quantities can be exciton density-dependent. For instance, the exciton blockade can lead to the reduction of G_n, and the scattering between excitons broadens the linewidth γ_n. These effects will eventually renormalise the Rabi splitting or the shift of polariton energy which directly translates into optical nonlinearity. To see these effects, we expand Eq. (S6) at low exciton density ρ as

$$\Omega_n = \Omega_n^{(0)} - \beta_n\rho + \mathcal{O}(\rho^2), \tag{S7}$$

where the Rabi splitting in low density is

$$\Omega_n^{(0)} = 2\sqrt{G_n^{(0)}\Lambda_n - \tfrac{1}{4}(\gamma_n^{(0)})^2}, \tag{S8}$$

with $G_n^{(0)} := G_n|_{\rho=0}$, $\gamma_n^{(0)} := \gamma_n|_{\rho=0}$, and $\Lambda_n := \sqrt{\frac{1}{2}\gamma_n^{(0)}(\kappa+\gamma_n^{(0)}) + (G_n^{(0)})^2}$. Here, the β-factor then reads

$$\beta_n = -\frac{2}{\Omega_n^{(0)}\Lambda_n}\Big[(\Lambda_n^2 + (G_n^{(0)})^2)\frac{dG_n}{d\rho} + G_n^{(0)}(\tfrac{1}{2}\kappa + \gamma_n^{(0)})\frac{d\gamma_n}{d\rho}\Big]. \tag{S9}$$

This factor quantifies the rate of the Rabi splitting reduction. In the above, the exciton blockade and the linewidth broadening effects are present in the first and second terms. However, no strong inhomogeneous broadening has been resolved in the measurement within the low-density regime. Therefore, in our analysis, we focus on the blockade effect in the first term.

7.2 Rydberg and Pauli blockade

In this subsection, we discuss the possible blockade mechanism that leads to reduction of G_n, and present the details for derivations. First, let us comment on the case of Rydberg excitons outside of optical cavities. In the presence of N Rydberg excitons, the absorption (α) of Cu_2O follows the scaling relation $\alpha \propto V/V_{\mathrm{B}} - N$, where V is the total volume of the system and V_{B} is the Rydberg blockade volume [13]. The N-dependent behavior in α can be well explained by Rydberg blockade physics [14]. When placed inside an optical cavity, the absorption is related to the light-matter coupling constant as $\alpha \propto G_n^2$ [1]. In the low-density limit, the coupling constant may be written as

$$G_n \approx G_n^{(0)}(1 - \tfrac{1}{2}B_nN), \tag{S10}$$

where $B_n = V_{\mathrm{B}}/V$ is the blockade coefficient for a single Rydberg exciton. In the case of Rydberg blockade it is given by

$$B_n = \frac{4\pi}{3}\frac{r_C^3}{V}. \quad \text{(Rydberg)} \tag{S11}$$

The Rydberg exciton blockade radius is modeled by $r_C = (C_k/\gamma_n)^{1/k}$ with C_k being the dipole-dipole interacting constant [14, 15]. Here, $k = 3$ is the Förster-type interaction and $k = 6$ is the van der Waals interaction. This parameter plays a crucial role in determining blockade physics which has been estimated theoretically in Ref. [15]. To calculate the β-factor, we substitute Eq. (S10) into Eq. (S9), and get

$$\beta_n = \frac{\Lambda_n^2 + (G_n^{(0)})^2}{\Omega_n^{(0)}\Lambda_n} G_n^{(0)} B_n V. \tag{S12}$$

We then extract the light-matter coupling constant $G_n^{(0)}$ from the measurement in Fig. 3**a** of the main text by using Eq. (S8). This gives the best fit $G_n^{(0)} = 2.29(\frac{n^2-1}{n^5})^{1/2}$meV in the main text, see Fig. **S6a**. We obtain the β-factor

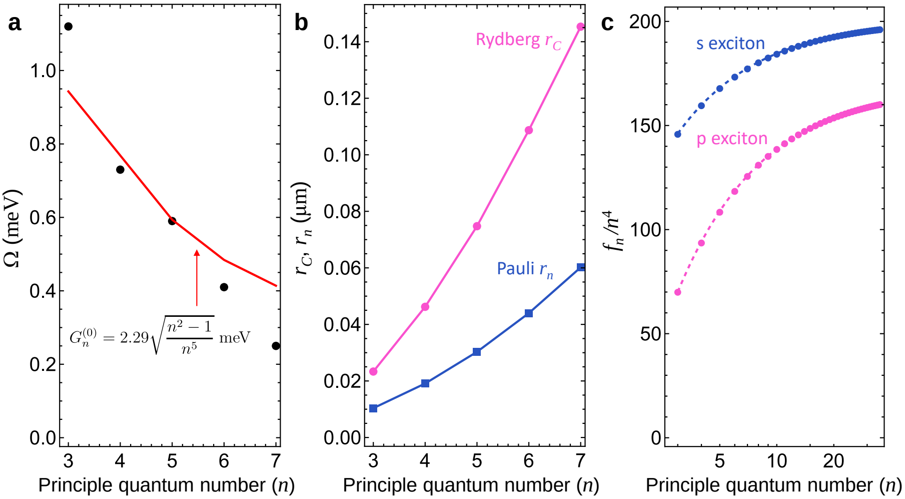

Fig. S6: Scaling of Rydberg exciton. (a) Rabi splitting in low density. Closed black circles are the point taking from Fig. 3**a** of the main text at $\rho = 0$. Dashed curved is the fitted result by using $G_n^{(0)} \propto \sqrt{(n^2-1)/n^5}$ and Eq. (S6). (b) Comparison between Rydberg (r_C magenta) and Pauli (r_n, light blue) blockade radius. (c) Scaling of Pauli blockade coefficient. The factor f_n in Eq. (S18) gives the asymptotic scaling behavior for the blockade coefficient $B_n \sim n^4$ [Eq. (S17)]. Dashed curves are the fits by using $f_n = n^4(b_0 + b_1 n^{-1} + b_2 n^{-2})$.

plotted as the purple solid curve in Fig. 3**b**. In the large n-limit, the blockade coefficient shows a power-law scaling with $B_n \sim n^7$[14]. Hence, Eq. (S12) gives the asymptotic scaling for $\beta_n \sim n^{5.5}$.

However, this scaling property which is very often used for identifying the Rydberg blockade has only been established in the large quantum number regimes ($n \geq 12$) [14]. In our case, the exciton quantum number are in the range from $n = 3$ to $n = 7$, where the scaling behavior may not be evident. Therefore, the power-law scaling may not be a single argument supporting the observation of the Rydberg blockade in our low-n measurement. Next, we consider another potential contribution to the reduction of Rabi frequency.

As the exciton radius $r_n = \frac{1}{2}a_0(3n^2 - 2)$ [14] is comparable to the Rydberg blockade radius r_C (see Fig. **S6**b), we consider the effects of Pauli blockade. This comes from the composite nature of excitons and fermionic statistics of the electrons and holes. In order to identify the Rydberg physics in this low-n regime, we analyse the Pauli blockade and investigate its contribution.

For the Pauli blockade, the coefficient in Eq. (S10) can be evaluated exactly as

$$B_n = \sum_{\boldsymbol{k}} |\psi_n(\boldsymbol{k})|^4, \quad \text{(Pauli)}, \tag{S13}$$

where $\psi_n(\boldsymbol{k})$ is the exciton wavefunction with quantum number n and wavevector $\boldsymbol{k}$ [16]. The p-wave hydrogen-like wavefunction [17] ($l = 1, m = 0$)

is

$$\psi_n(\boldsymbol{k}) = \sqrt{\frac{(na_0)^3}{V}\frac{n(n-l-1)!}{(n+l)!}}\left[C_{n-l-1}^{(l+1)}(\tfrac{\xi^2-1}{\xi^2+1})\frac{2^{2l+3}2\pi l!\xi^l}{(\xi^2+1)^{l+2}}\right]\Upsilon_{lm}(\theta,\phi), \quad \text{(S14)}$$

where $\xi = nka_0$. Here, $C_n^{(\alpha)}(x)$ is the Gegenbauer functions and $\Upsilon_{lm}(\theta,\phi)$ is the spherical harmonic function. We note that the momentum $\boldsymbol{k}$ is discrete, defined by a finite sample size with volume V. The normalisation condition is $\sum_{\boldsymbol{k}} |\psi_n(\boldsymbol{k})|^2 = 1$. In contrast to the Rydberg blockade, the shape of the exciton wavefunction $\psi_n(\boldsymbol{k})$ completely determines B_n or the Bohr radius a_0. The Bohr radius can be determined by the experiment's Rydberg exciton energies, $\omega_n = E_{\rm g} + E_n^{\rm b}$ in Fig. **S3** with bandgap energy $E_{\rm g}$ and the exciton binding energy

$$E_n^{\rm b} = -\frac{\rm Ry^*}{n^2}. \quad \text{(S15)}$$

The Rydberg constant $\mathrm{Ry}^* = \frac{e^2}{4\pi\epsilon_0\epsilon_r}\frac{1}{2a_0}$, which allows us to estimate a_0. Using the Cu_2O dielectric constant $\epsilon_r = 7.5$ [18], and

$$\mathrm{Ry}^* = -\frac{\omega_{n-1}-\omega_n}{(n-1)^{-2}-n^{-2}}, \quad \text{(S16)}$$

we can deduce the Bohr radius $a_0 \approx 0.83$ nm. Therefore, in the Pauli blockade, the experiment leaves no free adjustable parameter for the β-factor. We plot β_n from the contribution due to the Pauli blockade in Fig. 3 of the main text (blue dashed curve). It is an order of magnitude smaller than the measured values.

In terms of power-law scaling, we let

$$B_n = f_n\frac{a_0^3}{V}. \quad \text{(Pauli)} \quad \text{(S17)}$$

The prefactor is

$$f_n = \frac{9\times 2^{20}n^3}{10(n^2-1)^2}\int_0^\infty d\xi\frac{\xi^6[C_{n-2}^{(2)}(\frac{\xi^2-1}{\xi^2+1})]^4}{(1+\xi^2)^{12}}, \quad \text{(S18)}$$

where we used $\sum_{\boldsymbol{k}}\frac{(2\pi)^3}{V} \to \int d^3k$ for wavevector $\boldsymbol{k}$ in large V. This f_n prefactor determines the scaling behavior of B_n and we plot in Fig. **S6**c. As we can see, the Pauli blockade coefficient (B_n) scales with a power law weaker than n^4 for low n, and approaches an $\sim n^{3.5}$ scaling in the high-n range. Overall, it is lower than the Rydberg blockade scaling with n^7. Using Eq. (S12), this yields a a scaling trend of $\beta_n \sim n^{2.5}$ for small n, significantly different as compared to experiment.

7.3 $\mathrm{n_2}$ parameter from Rabi frequency measurement

To estimate the $\mathrm{n_2}$ nonlinear parameter, we begin with the definition of the total refractive index and the optical susceptibility as follows [4]

$$\mathrm{n_T}^2 = \epsilon_\mathrm{b} + \chi(\omega). \tag{S19}$$

The optical susceptibility in a cavity for each excitonic mode near the resonance can be modeled by [1, 11]

$$\chi(\omega) \approx \frac{h_n G_n^2}{\omega - \omega_n + \frac{1}{2} i\gamma_n}, \quad (\omega \simeq \omega_n), \tag{S20}$$

where ω_n is the Rydberg exciton energy. We note that the constant of proportionality h_n can be determined from the Rabi splitting measurement.

The coupling constant G_n changes due to the Rydberg blockade as the laser power increases [Eq. (S10)]. This leads to the nonlinear response in the susceptibility

$$\chi(\omega) = \chi^{(1)}(\omega) + \chi^{(3)}(\omega) E^2. \tag{S21}$$

In the vicinity of $\omega \simeq \omega_n$, the linear response of the above is $\chi^{(1)}(\omega) \approx h_n (G_n^{(0)})^2 (\omega - \omega_n + \frac{1}{2} i\gamma_n)^{-1}$, and the Kerr nonlinear response is

$$\chi^{(3)}(\omega) \approx \frac{h_n (G_n^{(0)})^2 (-B_n)(\frac{1}{2}\epsilon_0 V/\omega)}{\omega - \omega_n + \frac{1}{2} i\gamma_n}, \tag{S22}$$

where we have converted the exciton number N into the electric field E by using $\omega N/V \approx \frac{1}{2}\epsilon_0 E^2$ with V being the volume of the nonlinear medium. Also, the blockade coefficient is given by Eq. (S11). The nonlinear refractive index $\mathrm{n_2}$ is defined as

$$\mathrm{n_2}(\omega) = \frac{\mathrm{Re}[\chi^{(3)}(\omega)]}{\epsilon_0 c \mathrm{n}_0^2}, \tag{S23}$$

where $\mathrm{n}_0^2 = \epsilon_\mathrm{b} + \mathrm{Re}[\chi^{(1)}(\omega)]$. Therefore, the $\mathrm{n_2}$-parameter (near $\omega \simeq \omega_n$) from the quench of Rabi frequency is

$$\mathrm{n_2}(\omega) \approx -\frac{h_n \beta_n}{2c\mathrm{n}_0^2 \omega} \frac{G_n^{(0)}(\omega - \omega_n)}{(\omega - \omega_n)^2 + \frac{1}{4}\gamma_n^2}, \tag{S24}$$

where we have used Eq. (S12) by taking the strong-coupling limit ($G_n^{(0)} \gg \gamma_n^{(0)}$).

At the polaritonic peaks ($\omega = \omega_n \pm \frac{1}{2}\Omega_n^{(0)}$), the total refractive index satisfies [11]

$$\mathrm{n_T}(\omega_n \pm \tfrac{1}{2}\Omega_n^{(0)}) = \mathrm{n_b} \frac{\omega_n}{\omega_n \pm \frac{1}{2}\Omega_n^{(0)}} \tag{S25}$$

where $n_b = n_T(\omega_n) = \sqrt{\epsilon_b}$ is the background refractive index ($n_b = 1$ for the device is vacuum). The condition in Eq. (S25) determines the constant of proportionality h_n. Therefore, we can deduce the nonlinear refractive index n_2 from our Rabi splitting measurements.

Alternatively, we can also deduce the constant of proportionality h_n from the absorption data by following the method in Ref. [11]. First, the Rabi splitting of a nonlinear medium with susceptibility in Eq. (S20) can also be estimated as

$$\Omega_n^{(0)} = \sqrt{2\omega_n h_n (G_n^{(0)})^2/{n_b}^2 - (\gamma_n^{(0)})^2} \tag{S26}$$

Substituting the experimental measurements into the above, we get h_n which has the same orders of magnitude as the h_n obtained from Eq. (S25). Furthermore, in Ref.[11], we have $h_n(G_n^{(0)})^2 \approx (n_b c\alpha_0\gamma_n)/(\omega_n/\hbar)$ where α_0 is the absorption. Using the absorption data in Ref. [13], we again obtain the h_n with the same order of magnitude.

8 Non-resonant pumping and quenching of Rabi Splitting

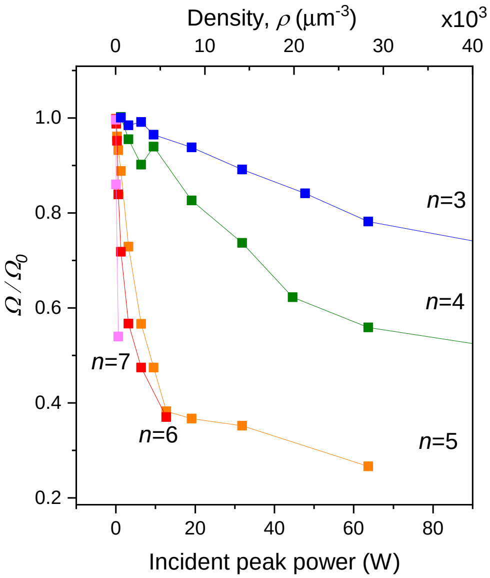

Fig. S7: Normalized Rabi splitting as a function of resonant peak laser power (bottom axis) and/or photon density in the cavity (top axis). Normalization is based on the case where smallest value of laser power is used.

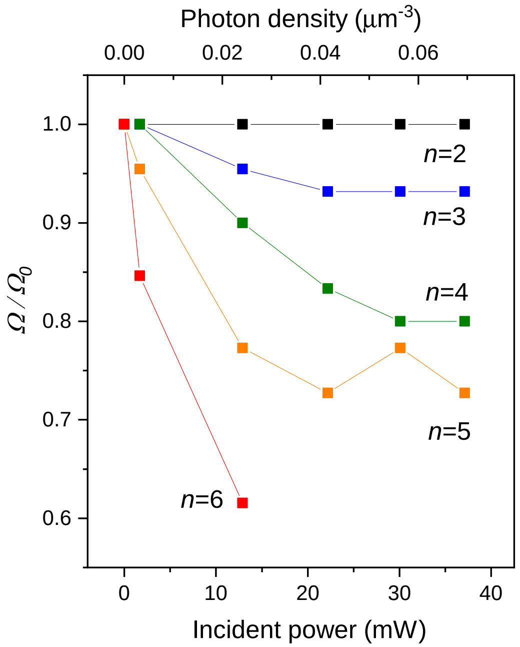

Fig. S8: Normalized Rabi splitting as a function of non-resonant CW laser power (bottom axis) and/or photon density inside the cavity (top axis). Rabi splitting values are normalized based on the case where no non-resonant laser is used.

In this section we compare two regimes of quenching of Rabi splitting. One case is the resonant pulsed excitation presented in the main paper in Fig. 3. Here we show these data as normalised Rabi splitting plotted as a function of incident peak power and/or photon density created by the pulse in the active cavity region (Fig. **S7**). The second case is non-resonant continuous wave (CW) excitation with above band gap green laser at wavelength 520 nm (Fig. **S8**). Varying the power of non-resonant laser and probing with weak broadband super-continuum laser filtered from 568 to 582 nm in transmission geometry the transmission spectra are recorded and then fitted to extract Rabi splitting. Normalised Rabi splitting for this experiment is plotted as a function of CW power of green laser or photon density in the cavity for excitons from $n = 2$ to 6 (see Fig. **S8**).The photon density in pulsed case is calculated using Eq. S2 whereas photon density in CW case is calculated using the following equation:

$$\rho^{\mathrm{CW}} = \frac{TP_{inc}}{3A\hbar\omega(c_0/n_0)}, \tag{S27}$$

where T is the transmission through the mirror(2%), P_{inc} is the incident power, A is the illuminated area (95 μm^2), $\hbar\omega$ is the photon energy, c_0 is the speed of light in vacuum, n_0 is Cu_2O background refractive index, 1/3 is from the

fact that propagation length of the excitation laser ≈ 10 μm in Cu_2O with thickness of ≈ 30 μm. The data for CW case are obtained on microcavity Cu_2O sample with distributed Bragg reflector mirrors reported in Ref. [1], which is very similar in optical characteristics to the sample presented in the main paper — cavity with silver mirrors. Note that the incident powers used in CW excitation case are in tens of mW range for quenching of Rabi splitting whereas in resonant experiment with pulsed excitation quenching of Rabi splitting is achieved at peak powers of tens of Watts. Even higher difference can be noted in the photon density required for quenching the Rabi between CW and pulsed regime (6 orders of magnitude). Such big difference of 6 orders of magnitude in photon densities can be explained by population of free electrons and holes and long lived states in the case of CW excitation contributing to nonlinear behaviour and quenching of Rabi splitting. We also note that in our experiments the photon densities in case of non-resonant CW pumping are only 6 orders of magnitude less than in the case of pulsed excitation. So our experiment with CW nonresonant pumping alone cannot explain why there is 8 orders of magnitude difference in the n_2 parameters measured in the case of resonant pulsed and CW pumping in Ref. [4]. It is possible that such a difference is sample dependent (for example, the density of long-lived localised states, which could be associated with metallic impurities may vary from sample to sample).

9 Pump-probe zero delay point

The interference between residual pump and probe pulses results in modulation of the spectra at small delays between pulses (see Fig. **S9**a). The modulation frequency depends on separation of the pulses whereas its visibility depends on the relative intensities of the two pulses. The analysis of modulation at small delay times allow us to define the zero delay between pulses with accuracy of ±0.25 ps (see Fig. **S9**). Fig. **S9**a shows pump-probe transmission spectra on glass substrate without the Cu_2O at different delay times where pump signal after rejection with a polariser was ≈ 3 times bigger than the probe signal. We extract free spectral range (FSR) of the modulated signal and plot it as a function delay stage position in Fig. **S9**b. Fitting the FSR data allow us to define the zero delay position for the probe delay stage.

Although we have used much smaller pump powers in the experiment with Cu_2O in Fig. 5 and rejected the unwanted pump signal with polarisers on detection small amount of pump still provides enough modulation for the probe signal to interfere with polariton resonance. So we not plot data points in Fig. 5 of the main text for the range of -30 to 37 ps apart from exact time 0 where the frequency of modulation is bigger than the polariton resonance and it doesn't influence the transmitted probe polariton spectrum.

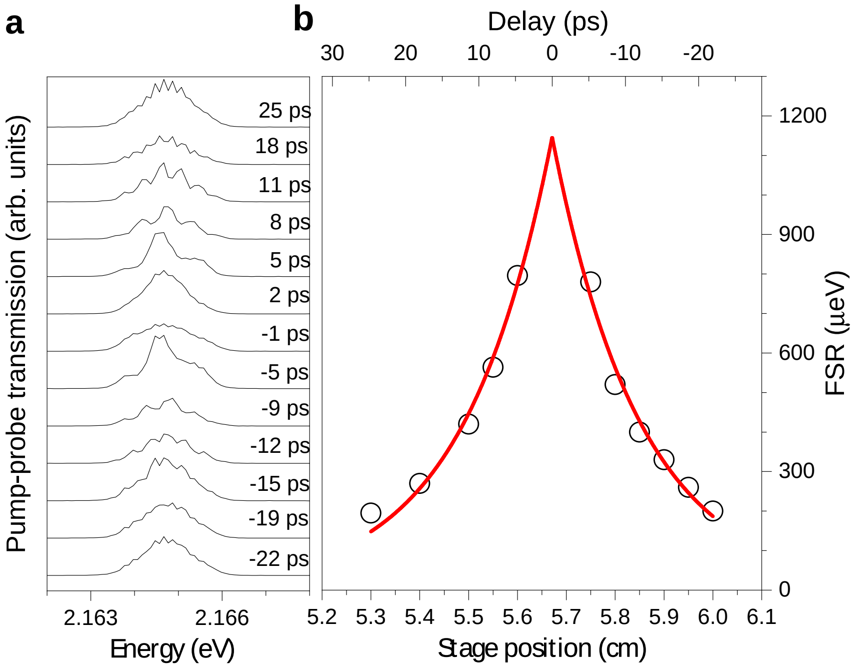

Fig. S9: **Pump-probe signal on substrate without the sample.** **a** transmitted probe spectra for different delays displays interference with residual pump beam not fully rejected by polarisers. Intensity of probe (pump) beam is 20 μJ cm^{-2} (20 mJ cm^{-2}). Period of modulation in energy or free spectral range (FSR) increases closer to zero delay. **b** FSR plot (extracted from spectra in **a**) as a function of probe delay stage position (bottom axis) and/or time delay (top axis). Time zero between arrival of two pulses is defined from the fit of FSR with exponential function with accuracy of ± 0.25 ps.

References

[1] Orfanakis, K., Rajendran, S.K., Walther, V., Volz, T., Pohl, T., Ohadi, H.: Rydberg exciton–polaritons in a Cu_2O microcavity. Nature Materials **21**(7), 767–772 (2022). https://doi.org/10.1038/s41563-022-01230-4

[2] Kuriakose, T., Walker, P.M., Dowling, T., Kyriienko, O., Shelykh, I.A., St-Jean, P., Zambon, N.C., Lemaître, A., Sagnes, I., Legratiet, L., Harouri, A., Ravets, S., Skolnick, M.S., Amo, A., Bloch, J., Krizhanovskii, D.N.: Few-photon all-optical phase rotation in a quantum-well micropillar cavity. Nature Photonics **16**(8), 566–569 (2022). https://doi.org/10.1038/s41566-022-01019-6

[3] Ferrera, M., Magnozzi, M., Bisio, F., Canepa, M.: Temperature-dependent permittivity of silver and implications for thermoplasmonics. Phys. Rev.

Mater. **3**, 105201 (2019). https://doi.org/10.1103/PhysRevMaterials.3.105201

[4] Morin, C., Tignon, J., Mangeney, J., Dhillon, S., Czajkowski, G., Karpiński, K., Zielińska-Raczyńska, S., Ziemkiewicz, D., Boulier, T.: Self-kerr effect across the yellow rydberg series of excitons in Cu_2O . Physical Review Letters **129**(13) (2022). https://doi.org/10.1103/physrevlett.129.137401

[5] Carusotto, I., Volz, T., Imamoğlu, A.: Feshbach blockade: Single-photon nonlinear optics using resonantly enhanced cavity polariton scattering from biexciton states. EPL (Europhysics Letters) **90**(3), 37001 (2010). https://doi.org/10.1209/0295-5075/90/37001

[6] Walker, P.M., Tinkler, L., Skryabin, D.V., Yulin, A., Royall, B., Farrer, I., Ritchie, D.A., Skolnick, M.S., Krizhanovskii, D.N.: Ultra-low-power hybrid light–matter solitons. Nature Communications **6** (2015). https://doi.org/10.1038/ncomms9317

[7] Ferrier, L., Wertz, E., Johne, R., Solnyshkov, D.D., Senellart, P., Sagnes, I., Lemaître, A., Malpuech, G., Bloch, J.: Interactions in confined polariton condensates. Phys. Rev. Lett. **106**, 126401 (2011). https://doi.org/10.1103/PhysRevLett.106.126401

[8] Estrecho, E., Gao, T., Bobrovska, N., Comber-Todd, D., Fraser, M.D., Steger, M., West, K., Pfeiffer, L.N., Levinsen, J., Parish, M.M., Liew, T.C.H., Matuszewski, M., Snoke, D.W., Truscott, A.G., Ostrovskaya, E.A.: Direct measurement of polariton-polariton interaction strength in the thomas-fermi regime of exciton-polariton condensation. Phys. Rev. B **100**, 035306 (2019). https://doi.org/10.1103/PhysRevB.100.035306

[9] Brichkin, A.S., Novikov, S.I., Larionov, A.V., Kulakovskii, V.D., Glazov, M.M., Schneider, C., Höfling, S., Kamp, M., Forchel, A.: Effect of coulomb interaction on exciton-polariton condensates in gaas pillar microcavities. Phys. Rev. B **84**, 195301 (2011). https://doi.org/10.1103/PhysRevB.84.195301

[10] Fieramosca, A., Polimeno, L., Ardizzone, V., Marco, L.D., Pugliese, M., Maiorano, V., Giorgi, M.D., Dominici, L., Gigli, G., Gerace, D., Ballarini, D., Sanvitto, D.: Two-dimensional hybrid perovskites sustaining strong polariton interactions at room temperature. Science Advances **5**(5) (2019). https://doi.org/10.1126/sciadv.aav9967

[11] Khitrova, G., Gibbs, H.M., Jahnke, F., Kira, M., Koch, S.W.: Nonlinear optics of normal-mode-coupling semiconductor microcavities. Rev. Mod. Phys. **71**, 1591–1639 (1999). https://doi.org/10.1103/RevModPhys.71.1591

[12] Savona, V., Andreani, L.C., Schwendimann, P., Quattropani, A.: Quantum well excitons in semiconductor microcavities: Unified treatment of weak and strong coupling regimes. Solid State Communications **93**(9), 733–739 (1995). https://doi.org/10.1016/0038-1098(94)00865-5

[13] Kazimierczuk, T., Fröhlich, D., Scheel, S., Stolz, H., Bayer, M.: Giant rydberg excitons in the copper oxide Cu_2O. Nature **514**(7522), 343–347 (2014). https://doi.org/10.1038/nature13832

[14] Aßmann, M., Bayer, M.: Semiconductor rydberg physics. Advanced Quantum Technologies **3**(11), 1900134 (2020). https://doi.org/10.1002/qute.201900134

[15] Walther, V., Krüger, S.O., Scheel, S., Pohl, T.: Interactions between rydberg excitons in Cu_2O. Phys. Rev. B **98**, 165201 (2018). https://doi.org/10.1103/PhysRevB.98.165201

[16] Combescot, M., Betbeder-Matibet, O., Dubin, F.: The many-body physics of composite bosons. Physics Reports **463**(5-6), 215–320 (2008)

[17] Podolsky, B., Pauling, L.: The momentum distribution in hydrogen-like atoms. Phys. Rev. **34**, 109–116 (1929). https://doi.org/10.1103/PhysRev.34.109

[18] Kavoulakis, G.M., Chang, Y.-C., Baym, G.: Fine structure of excitons in Cu_2O. Phys. Rev. B **55**, 7593–7599 (1997). https://doi.org/10.1103/PhysRevB.55.7593
